# Brentuximab-Induced Peripheral Neurotoxicity: A Multidisciplinary Approach to Manage an Emerging Challenge in Hodgkin Lymphoma Therapy

**DOI:** 10.3390/cancers13236125

**Published:** 2021-12-05

**Authors:** Roser Velasco, Eva Domingo-Domenech, Anna Sureda

**Affiliations:** 1 Neuro-Oncology Unit, Department of Neurology, Hospital Universitari de Bellvitge-Institut Català dOncologia, 08908 Barcelona, Spain; 2 Department of Cell Biology, Physiology and Immunology, Institute of Neurosciences, 08193 Bellaterra, Spain; 3 Department of Haematology, Catalan Institute of Oncology, Hospital Duran i Reynals, IDIBELL, University of Barcelona (UB), L’Hospitalet de Llobregat, 08908 Barcelona, Spain; edomingo@iconcologia.net (E.D.-D.); asureda@iconcologia.net (A.S.)

**Keywords:** Hodgkin lymphoma, brentuximab vedotin, neurotoxicity, peripheral neuropathy, chemotherapy-induced peripheral neuropathy, multidisciplinary

## Abstract

**Simple Summary:**

This article highlights the importance of monitoring peripheral neurotoxicity in patients with Hodgkin lymphoma (HL) who receive brentuximab vedotin, with a particular focus on early recognition and multidisciplinary management of this type of toxicity. Brentuximab vedotin-induced neurotoxicity (BVIN) is the most common non-haematological complication and cause of dose-delay or early discontinuation of BV treatment. An accurate and prompt diagnosis in patients with HL is essential to optimise management of this potentially disabling complication.

**Abstract:**

Brentuximab vedotin (BV) is an anti-CD30 antibody–drug conjugate approved to treat classical Hodgkin lymphoma (HL). BV-induced peripheral neurotoxicity (BVIN) is one of the greatest concerns for haematologists treating HL for several reasons. First, BVIN is highly frequent. Most patients receiving BV will experience some degree of BVIN, resulting in the primary reason for dose modification or discontinuation of HL therapy. Second, BV produces sensory, motor, and/or autonomic peripheral nerve dysfunction, which can present as severe, disabling forms of BVIN—predominantly motor—in some patients. Third, although largely reversible, BVIN may persist months or years after treatment and thereby become a major issue in HL survivorship. BVIN may, therefore, negatively affect the quality of life and work-life of often young patients with HL, in whom long-term survival is expected. Currently, the only strategy for BVIN includes dose adjustments and treatment discontinuation; however, this could interfere with LH therapy efficacy. In this setting, early recognition and adequate management of BVIN are critical in improving clinical outcomes. Careful neurologic monitoring may allow accurate diagnoses and gradation of ongoing forms of BVIN presentation. This review analysed current, available data on epidemiology, pathophysiology, patient- and treatment-related risk factors, clinical and neurophysiologic phenotypes, and management in patients with HL. Furthermore, this review specifically addresses limitations posed by BVIN assessments in clinical practice and provides skills and tools to improve neurologic assessments in these patients. Integrating this neurotoxic drug in clinical practice requires a multidisciplinary approach to avoid or minimise neurotoxicity burden in survivors of HL.

## 1. Introduction

Hodgkin lymphoma (HL) is a haematological malignancy that accounts for about 10% of all lymphomas [1,2]. Although most patients with HL are cured with initial therapy, up to 20–30% of patients with advanced HL are refractory to initial therapy or will relapse (R/R HL), requiring further therapeutic options [2]. Classical HL (cHL) is the most frequent type of HL. It is histopathologically defined by the presence of malignant Hodgkin Reed–Sternberg cells that express the CD30, a surface antigen. Brentuximab vedotin (BV) (Adcetris^®^, Seagen, Inc., Bothell, WA, USA) is the first anti-CD30 antibody-drug conjugate (ADC) to be approved by both the Food and Drug Administration (FDA) and European Medicines Agency (EMA) to treat patients with R/R HL [3]. BV has received approval for different cHL treatment settings, including newly diagnosed patients [4,5], and as consolidation treatment after autologous stem cell transplantations (ASCT) [6,7]. In addition to cHL, BV has shown to be highly effective in other CD30-positive neoplasms, such as peripheral T-cell lymphomas (PTCL), systemic anaplastic large cell lymphoma (sALCL), primary cutaneous anaplastic large cell lymphoma (pcALCL), and CD30-expressing mycosis fungoides (MF) [8,9,10,11]. Additionally, there have been multiple recent publications regarding the drug’s utility in other lymphoid malignancies, including diffuse large B-cell lymphoma (DLBCL), primary mediastinal lymphoma (PMBL), and post-transplant lymphoproliferative disorders (PTLD) [12].

However, peripheral neurotoxicity (PN) is a type of off-target toxicity of BV and represents the most common extra-haematological and the main clinically significant BV-related toxicity.

Although PN is not a new issue in HL treatment, BV-induced neurotoxicity (BVIN) has emerged as a clinical challenge. BVIN onset has become highly frequent, and many patients receiving BV will experience some degree of BVIN. As a result, BVIN accounts for the primary cause of treatment delay, dose modification, and early BV discontinuation [1,13]. BVIN may, therefore, affect therapy planning and dosage, which can later influence outcome of patients with HL. Similarly, BVIN symptoms may negatively affect patients’ lives, including domestic, work, social, and leisure activities, and thereby decrease quality of life. As most patients with HL will survive many years, BVIN also raises concerns in relation to cancer survivorship. BVIN could incur a significant increase in annual healthcare utilisation and costs, which has been observed elsewhere [14], as well as in patients with myeloma [15]. Therefore, PN constitutes an important clinical consideration, given its potential role in limiting prolonged administration of BV.

In the present review, we intended to summarise current knowledge on BVIN in HL treatment, both as a single agent and in combination, and direct particular attention to recognising and managing this complication to improve the multidisciplinary assessment of patients with HL.

## 2. Epidemiology of BVIN

BVIN can occur in patients with HL receiving BV as either a single agent in cases of R/R cHL [3] or as consolidation therapy after ASCT [6,7], or in combination with chemotherapy as salvage therapy before ASCT [16,17] or in newly diagnosed HL patients [4,5]. Reported overall rates of PN in adult patients with R/R HL range from 9 to 73% (Table 1). In phase II and III trials of BV monotherapy in HL, PN was reported in up to 67% of patients, with 20% of such events as grade 2 and 11–14% grade 3 [3,6]. In the AETHERA trial, in which BV monotherapy was administered up to 16 cycles, PN led to BV dose modification in nearly one-third of patients and to BV discontinuation in 23% [6]. Additionally, high rates of PN have also been observed in newly diagnosed scenarios. In ECHELON-1—a randomised, open-labelled, controlled trial in frontline therapy in advanced HL where BV in combination with AVD was compared to ABVD—PN was observed in 67% of patients, with a high incidence of moderate to severe events (20% grade 2, 11% grade 3) [4,5]. Of note, the rate of PN in the control arm (vinblastine-containing regimen without BV) of this trial was 43%, with 2% as grade 3, suggesting a cumulative/summatory effect of a two-agent combination with a known neurotoxic profile. Moreover, BV duration in combination trials was less than that in trials using BV as a single agent. In a recent multicentre study of BV + AVD × 4 cycles in newly diagnosed advanced HL, 54% of patients developed PN [18]. Conversely, PN was not a major issue during pre- and post-transplant phases in a separate multicentre phase I-II trial that included 66 patients with R/R HL after first-line chemotherapy who received a combination of BV and ESHAP. Amongst these patients, PN appeared in only three (5%) cases (grade 2) [17]. When BV is administered in combination with other non-neurotoxic agents, no substantial additional PN was observed. For example, in the ECHELON-2 phase III trial of patients with CD30-positive peripheral T-cell lymphomas, PN rates were similar between those receiving vincristine-containing regimen (CHOP) and those in the BV-containing arm (BV-CHP) (41% and 45% of patients, respectively) [19]. Finally, although BV dosing was higher in the ECHELON-2 trial (1.8 mg/kg/Q3W) than in that of ECHELON-1 (1.2 mg/Kg on days 1 and 15 every 28 days), the larger cumulated dose and higher number of BV doses in the latter were associated with an overall higher rate of PN.

In a real-life context, reported neurotoxicity rates in BV monotherapy in R/R HL are slightly lower to those published in clinical trial data, with grade ≥3 PN observed in 3.3-7.3% of cases [20] (Table 1). Importantly, overall higher rates of PN are observed when a formal, detailed neurologic assessment is performed. In a small clinical study including 10 patients with R/R HL receiving BV who had a detailed, prospective neurologic and neurophysiologic assessment during treatment, the authors identified that after a median of only four cycles, almost all patients (90%) developed new or progressive PN symptoms [21]. Of note, patients undergo a shorter number of cycles of BV in real-life practice than the median of BV cycles administered in clinical trials. Conversely, patients with R/R HL received a median of nine cycles of BV in a pivotal phase II study [3], whilst the median number of BV cycles was five in a retrospective real-world study [22]. Therefore, PN, especially low-grade PN, may have been under-reported in studies outside of these registration trials, given the retrospective nature of data derived from real-world patient files; data collection methodology, with investigators not identifying PN prospectively through monitored, direct examinations of patients; and data recording at the time of the studies [22,23].

PN can also be observed in standard HL treatment outside of BV-containing regimens due to other neurotoxic chemotherapy drugs. Initial chemotherapy in a newly diagnosed case of advanced-stage cHL typically involves ABVD (adriamycin, bleomycin, vinblastine plus dacarbazine), BEACOPPescalated (bleomycin, etoposide, adriamycin, cyclophosphamide, vincristine, procarbazine, prednisone, and granulocyte colony-stimulating factor), or Stanford V (doxorubicin, vinblastine, vincristine, bleomycin, etoposide, cyclophosphamide (or mechlorethamine or ifosfamide), prednisone) [62,63]. Paediatric patients often undergo treatment with ABVE-PC (doxorubicin, bleomycin, vinblastine, etoposide, prednisone, cyclophosphamide). Furthermore, standard second-line therapies for these patients include combination chemotherapy regimens such as ICE (ifosfamide, carboplatin, etoposide), DHAP (dexamethasone, high-dose cytarabine, cisplatin), GDP (gemcitabine, dexamethasone, cisplatin), or GIFOX (gemcitabine, ifosfamide, oxaliplatin). All of these regimens contain vinca-alkaloid or platinum agents known to produce PN to some degree. For example, the classical ABVD schedule can be associated with neuropathy in overall and grade 2-3 PN, up to 56% and 12%, respectively [64,65,66]. In addition, novel therapies such as immune checkpoint inhibitors such as anti-PD1 antibodies (i.e., nivolumab and pembrolizumab), which received recent approval for relapsed/refractory HL [67,68], can induce immune-mediated PN amongst other neurologic complications [69].

## 3. Mechanisms of Brentuximab-Induced Peripheral Neurotoxicity

ADCs allow high doses of cytotoxic drugs to be delivered to cancer cells, which are excessively toxic if not conjugated to monoclonal antibodies, whilst largely sparing exposure to healthy tissue. ADCs comprise three components: the antibody, linker, and payload. BV is an ADC that consists of a human chimeric immunoglobulin G1 antibody-directed against CD30, which is covalently linked to antitubulin agent monomethyl auristatin E (MMAE) through a protease-cleavable linker. Once BV binds to the extracellular domain of CD30 on Hodgkin Reed–Sternberg cells, it becomes internalised by clathrin-mediated endocytosis and subsequently travels to the lysosome for the linker to be cleaved by lysosomal proteases and MMAE released into the cytosol. MMAE binds to tubulin and inhibits microtubule (MT) polymerisation, inducing G2–M phase growth arrest and cell apoptosis [24,70]. Most ADC toxicity is often thought to be derived from the payload, which in this case would be MMAE [71].

Vedotin refers to MMAE plus its linking structure to the antibody. MMAE is a synthetic drug based on the auristatin structure, derived from a natural product called dolastatins [72]. Two main types of anti-microtubule drugs are commonly used in oncology: (1) those agents promoting tubulin polymerisation, including taxanes and ixabepilone, and (2) agents such as vinca alkaloids and eribulin that favour microtubule disassembly by inhibiting tubulin polymerisation. MMAE belongs to the latter group of microtubule-disrupting agents. The molecular mechanisms underlying normal MMAE actions as well as those governing MMAE-ADC-induced PN have been reported in in vitro models rather recently, showing that MMAE binds extensively to tubulin and MTs and causes severe MT dysregulation via blockade of tubulin polymerisation [73].

Neuropathy is an expected class effect of microtubule-binding drugs such as MMAE. Peripheral nerves are susceptible to MMAE-mediated inhibition of MT-dependent axonal transport due to axons’ long projections and the critical role of the MT network in maintaining long axonal transport between neuronal cell bodies and distal nerve endings [74]. Axonal degeneration can, therefore, occur and lead to PN of the most peripheral nerve terminals, beginning at the most distal extremities such as the fingertips and toes, and progressing proximally toward the trunk, given that the longest and largest axons may have the highest energy requirements and make them particularly vulnerable [73,74,75]. Pathologic findings in sural nerve biopsies of patients with severe PN have shown features including axonal neuropathy, Wallerian degeneration, and reduced MT density with a severe decrease in identifiable MT profiles in myelinated and, to a lesser extent, unmyelinated fibres [74].

Of note, PN has been consistently reported with all conventional MMAE ADCs, irrespective of target antigen [76,77]. Vedotin is used as a payload in other FDA-approved ADCs, including polatuzumab-vedotin (Polivy™) or enfortumab-vedotin (PadcevTM), to treat diffuse large B-cell lymphoma and metastatic urothelial carcinoma, respectively [71,78]. In a phase II study, polatuzumab-vedotin was associated with an incidence of grade ≥2 PN of 55–72% in patients with indolent non-Hodgkin lymphoma receiving a dosage of 1.8–2.4 mg/kg every three weeks until progression or for a maximum of 17 cycles [78]. The exact mechanisms of PN with vedotin-containing immunoconjugates remain unknown, and few investigations concerning the pathogenesis of PN are available. Interestingly, PN was not predicted in non-clinical toxicology studies performed in monkeys or rats treated with MMAE ADCs, limiting availability of knowledge on the pathophysiology of this complication [77].

In normal cells, CD30 expression is restricted to the thymic medulla and activated B and T lymphocytes [70]. Neurons do not seem to express CD30, as confirmed in pathologic data in the sural nerve biopsy of a patient with BVIN [79]. Therefore, BVIN is unlikely to be primarily driven by CD30-mediated uptake. The way by which MMAE reaches peripheral nerves remains unclear. MMAE is diffusible from CD30-positive lymphoma cells into the extracellular matrix and kills surrounding negative CD30-cells [62,70,80], although the extent of free MMAE exposure beyond the tumour microenvironment is unknown [70]. Furthermore, there is the possibility of MMAE (enzymatic) release and subsequent bystander cell killing without prior internalisation in CD30-positive cells [81]. Early cleavage of the linker, releasing free drug, may produce more widespread toxicities. In fact, MMAE conjugates with a protease-cleavable linker is less stable than other linkers, allowing for systemic release of free drug [71]. However, in vitro studies have indicated that MMAE was stably attached to the antibody (only 2% of MMAE was released from the ADC during 10 days of incubation in human plasma) prior to BV internalisation [62]. Small-molecule chemotherapy agents such as MMAE could enter cells via passive diffusion and might be able to be passively transported within peripheral nerve cells [77,82]. A long half-life might be an additional risk for ADC degradation and lead to non-specific toxicity of unconjugated MMAE [83]. Although detectable levels of unconjugated MMAE in blood have been observed, systemic unconjugated MMAE levels are typically very low; some authors argue that such levels are unlikely to contribute significantly to peripheral nerve exposure [77].

Furthermore, a potential immune basis of PN associated with BV has been suggested in some cases. BV might have the potential to induce or exacerbate inflammatory polyradiculoneuropathies [84]. Supporting evidence includes the presence of demyelination features in nerve conduction studies (NCS) with even predominant focal demyelination [84,85], and improvement with corticosteroids [85], plasma exchange, or intravenous immunoglobulin (IVIG) [84]. However, no inflammatory infiltrates in nerve biopsy were identified in one patient with severe PN due to BV [79]. The pathogenic mechanism responsible for peripheral nerve demyelination under BV remains unclear. Local extravasation of BV has been associated with focal neuropathy. One patient developed tingling paraesthesia, numbness, and difficulties in grasping with his right hand two weeks after partial extravasation in the setting of the median nerve with prominent demyelinating features in the NCS of the median nerve, which clinically resolved within eight weeks [82]. CD30 is a transmembrane glycoprotein receptor in the tumour necrosis factor (TNF)-receptor superfamily [86]. TNF-α inhibition has been associated with demyelinating neuropathies [87]. Fargeot et al. suggest that by targeting CD30, BV might have the potential to precipitate immune-mediated peripheral neuropathies, similar to TNF inhibitors [84]. Additionally, damage to myelin sheets and Schwann cells has also been documented in patients receiving microtubule agents [75].

## 4. Types and Forms of BVIN Presentation and Timing

BV may produce sensory, motor, and/or autonomic nerve dysfunction alone or in combination. BVIN is more frequently a sensory predominant syndrome involving large and/or small unmyelinated fibres. Patients typically complain of sensory symptoms including abnormal tactile perception (80%), vibratory sense (80%), numbness (70%), paraesthesia (70%), tingling (60%), and burning (40%), typically in a “sock-and-glove” distribution [21]. Hyperalgesia (exaggerated pain experience in response to a painful stimuli) and allodynia (experience of pain to a usually non-painful stimuli) can be present, even though the nature of BVIN is not always painful. Neuropathic pain was reported in 60% of cases with a low visual analogue scale (VAS) median peak of 2 (0–7, range), representing the most accurate characterisation of this type of neuropathy in patients with HL to date [21]. On physical examination, distal vibratory sensory loss and absence of myotatic reflexes are the most prominent findings [21,79]. Sensory ataxia can be observed in more affected patients [84], which is also predictive of a higher risk of falls in this population [88].

Prominent or early motor involvement has been reported in some patients. In a prospective study published by Mariotto et al., investigators described motor symptoms reported in half of the patients: distal upper limbs (10%), distal upper and lower limbs (30%), and distal upper limbs and diffuse lower limbs (10%) [21]. Loss of fine motor skills, myalgias and cramps are commonly seen with BV administration [21,74,85,89]. Weakness of foot dorsiflexion, steppage gait, muscle wasting, and fasciculations can be observed in severe cases [85]. Of note, motor BVIN usually occurs after or in conjunction with sensory symptoms. However, isolated presentation of this form two months before limited sensory complaints has been reported in one patient with severe PN, highlighting the fact that motor events can be followed by sensory manifestations in some cases [74]. Autonomic dysfunctions such as erectile dysfunction, constipation, and orthostatic hypotension are less frequently reported, affecting up to 10% of patients [21,90]. This figure may be underestimated, nonetheless. A rate of 12.5% of paralytic ileus was reported in one retrospective study including 30 patients with R/R HL and six patients with systemic ALCL [52].

**Figure 1:**
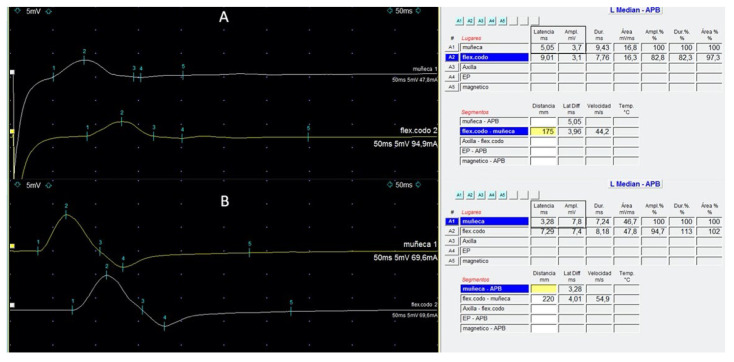
Left median motor nerve conduction studies at wrist (upper trace) and elbow (lower trace) in a patient with HL that developed grade-3 BVIN (**A**) and at recovery (**B**), 6 months later. The presence of delayed distal latencies (square in (**A**)) (normal < 3.9 ms); reduced CMAP amplitude (square in (A)) (normal > 6 mV) with preserved middle segment velocities, representing features of an acquired peripheral neuropathy with distal demyelination. Normalisation of distal latency and CMAP can be observed at recovery (**B**).

Nerve conduction studies provide mechanistic information relevant to BVIN pathophysiology, which correlates with clinical examination. The sensory nerve action potential (SNAP) provides information on the sensory nerve axon and its pathway from the distal receptors in the skin to the dorsal root ganglia, while the compound muscle action potential (CMAP) is an assessment of the motor nerve fibres from their origins in the anterior horn cell to their termination along muscle fibres. Various parameters of the SNAP and CMAP waveforms are used to determine the number of functioning nerve fibres and the speed of conduction [91]. Predominant axonal damage is typically identified [21,79]. Axonal (60%) or mixed (axonal and demyelinating, 40%) features of nerve damage were reported in the setting of sensory (40%) and sensory-motor neuropathy (60%) [21]. Typically, sensory action potentials are more affected than motor ones, with a relative sparing of sural nerves known as “sural sparing pattern” [21,84]. Of note, slowing conduction velocity and impairment of motor distal latencies (DLs) indicative of some degree of demyelination have been described [85]. For example, motor nerve conduction studies with a mild increase in distal motor latencies of median nerves, with severely reduced compound muscle action potential (Figure 1) and less affected ulnar nerves, can be observed [74]. Absence of the F waves was observed in severe cases presenting muscle strength impairment [85]. A needle electromyography study may show features of acute denervation and polyphasic motor unit potentials producing a less-than-full interference pattern [85], indicating the loss or less recruitment of motor units.

Neurologic symptoms due to BV can peak within the first several days after infusion and then wear off until or disappear by the next infusion. Conversely, the symptoms may increase in severity and duration and no longer resolve between cycles. The median timing of BVIN onset varies (Table 1), usually at three months after the first BV dose [92]. In a phase II study including 102 patients with R/R HL, patients treated with BV after auto-SCT had a median time to PN onset of 12.4 weeks; median time to onset of PN of grades 2 and 3 were 27.3 and 38 weeks, respectively [3]. In the phase III trial that included patients with HL at high risk of relapse or progression following auto-HCT, onset of PN was at a median of 14 (0.1–47) weeks, with PN of grade 2 at a median of 27 (0.4–52) weeks, and PN of grade 3 at a median of 34 (7–106) weeks [6]. In a cohort of patients with mycosis fungoides (MF), median time to onset for any PN was 15 (0.4–48) weeks (range after the first dose), whilst the median time to onset of PN of grade 2 was 20.8 (6–48) weeks [79]. Importantly, PN can be an extremely early adverse event and even occur after the first cycle.

As previously mentioned, BV treatment has been associated with inflammatory demyelinating polyradiculoneuropathies. Retrospective research across seven French neurology departments between 2013 and 2019 identified 11 patients, of whom 10 had a HL diagnosis. Most of these patients received prior exposure to neurotoxic agents, including vinca alkaloids (10 of 11, 91%); however, they did not have overt PN before BV initiation. After a median of 7 (2–12) cycles, 18%, 45%, and 36% developed acute, subacute, and chronic forms of PN, respectively, presenting as Guillain–Barré syndrome or “definite” chronic inflammatory demyelinating polyradiculoneuropathies (CIDPs) with predominant and significant demyelinating features in NCS. Ten (91%) patients developed mild-to-severe motor deficits, prevailing in (but not limited to) distal muscles. Eight (73%) patients required support for walking [84].

## 5. Risk Factors for BVIN

BVIN is a known cumulative adverse event [62,89]. The level and duration of MMAE exposure in the peripheral nerve tissue is believed to be the most important determinant of PN [77]. In pivotal trials, increases in exposure to ADC and free MMAE were approximately proportional to doses. In a phase I, dose-escalation trial on 12 patients with R/R HL, the maximum tolerated BV dose was determined to be 1.8 mg/kg every three weeks [24]. A greater incidence and shorter time to PN were observed with weekly regimens [25], perhaps due to the higher BV exposure in peripheral nerves and/or inadequate time for repair mechanisms due to more frequent dosing. In detail, when compared against BV administration every three weeks, weekly BV resulted in a higher rate of neuropathy (73% vs. 36% respectively, any grade), a shorter time to onset (6.1 vs. 9 weeks), and more cases of treatment discontinuation. As a result, infusion every three weeks was established as the standard dose [13,77]. As summarised in Table 1, higher rates of PN are observed in those studies in whom patients received a higher number of BV cycles.

It is still unclear as to whether individual patient factors including demographics and patient characteristics play a role in increasing susceptibility to BVIN [79]. HL incidence is highest in adolescents and young adults, although HL can affect elderly individuals. Unfortunately, these populations are under-represented (only 5–10% of the patients treated in current HL clinical trials are ≥60 years old) or absent from large, randomised therapy trials [93]. BV has been associated with PN in elderly patients both in monotherapy [90,94] and in combination regimens [95]. In the setting of BV as frontline therapy, a phase II trial evaluating single-agent BV in 27 elderly patients with HL, with a median age of 78 years (five patients aged > 85 years), who received a median of 8 (range 3–23 months) cycles of treatment, showed that most patients (89%) experienced treatment-emergent PN, with grade 3-PN events being relatively high (30% overall). This was particularly the case amongst patients with known risk factors such as diabetes and/or hypothyroidism (46% vs. 14% for those without such risk factors). Pre-existing neuropathy, however, did not appear to predispose the patients in this study to grade 3-PN onset [90]. The prospective single-arm, multicentre phase II BREVITY study with BV monotherapy in a U.K. population of 35 patients with untreated cHL who were elderly (median age 77 years), frail, or had significant comorbidities at any age reported that more than half of the patients had PN. Eight of these patients permanently stopped treatment due to PN [94]. Recently, results from older patients with cHL treated in a large phase III ECHELON-1 clinical trial of frontline BV plus +AVD versus ABVD including 181 patients evaluable for safety aged ≥ 60 years (A + AVD: *n* = 83, ABVD: *n* = 98) showed a higher rate of any grade PN (65% vs. 43%) and grade 3–4 PN (18% vs. 3%), with a median of six treatment cycles across both treatment arms. The rate of severe PN was also higher in older (18%) than younger (9%) patients [93]. Of note, ECHELON-1 was not powered for age-based subgroup analyses, and *p*-values were not reported. Beyond age, contribution to the burden of BVIN from potential factors including diabetes and alcohol consumption is unknown, mainly due to the younger nature of patients included in clinical trials. There are conflicting results regarding the role of diabetes mellitus as a risk factor of CIPN [96,97].

Currently, the majority of patients with HL have been previously treated with prior chemotherapy regimens before BV therapy [26], frequently including neurotoxic agents [64]. In a series of 36 patients with MF, a logistic regression analysis showed that the likelihood of developing clinically significant BVIN increased 13-fold (95% CI 2.59–65.20) in those patients who received treatments in the previous year [79]. Pre-existing PN rates in patients with HL ranged from 5 to 40% at the time of enrolment in clinical trials or studies [3,25,26,39,47,95]. In detail, clinically and neurophysiologically documented PN was present in 30% of cases prior to BV therapy [21]. In this study, PN progression during treatment was observed in all subjects with pre-existing PN [21]. Conversely, lack of worsening of pre-existing PN has also been reported. In a recently published clinical trial, grade 1-2 PN was present before study entry in 11 patients (*n* = 1, grade 2); however, it did not worsen during BV-DHAP treatment [39]. One patient with a previous diagnosis of demyelinating polyneuropathy did not experience any neuropathy deterioration during the treatment course [45]. Along this same line, no differences in BV tolerability were observed in patients with R/R sALCL presenting with or without pre-existing PN [10]. However, similar to vincristine administration, BV may trigger early and severe neuropathies in patients with a previously underlying hereditary neuropathy (often unknown) like Charcot–Marie–Tooth (CMT) demyelinating neuropathy [79,98,99]. To our knowledge, no data have been made available or reported regarding genes related to this type of neurotoxicity.

Besides pre-existing conditions or prior anti-cancer therapies, it has been found that concurrent medications or nutritional supplements (i.e., pyridoxine) or herbal preparations may affect BVIN onset. Importantly, strong CYP3A inhibitors may increase susceptibility to BVIN [98]. For example, administering BV with a strong CYP3A inhibitor (i.e., ketoconazole) increases MMAE exposure by approximately 34% [100]. However, the impact of this modest increase in MMAE exposure on the risk of PN is unknown due to the limited number of patients and treatment duration [77,100]. In summary, patients whose medical history includes those potential risk factors should be monitored during BV therapy. Further data would elucidate the exact role of these factors in BVIN onset.

## 6. Early Diagnosis and Management of BVIN

### 6.1. Early Detection and Timely Assessment of BVIN Are Crucial in Achieving a Better Outcome

BVIN may occur at any time throughout treatment; early detection of PN is critical. Patients should be educated on the need to report symptoms and signs of BVIN early to their healthcare team. Of note, underreporting and under-description of CIPN to the clinical team may occur [101,102]. A recent observational study in women with breast cancer treated with paclitaxel identified that women who were not working had a lower-income status and displayed higher trust in their oncologists’ competency were more likely to underreport CIPN symptoms, revealing such features as significant factors associated with under-description of CIPN to clinicians [102]. Patients with HL might also underreport or minimise their complaints due to the fear of their cancer being undertreated. This could thus increase the risk of death or even cause these patients to face difficulties in interpreting and describing sensations to their healthcare providers [103]. Therefore, clinicians should assess PN regularly during BV therapy to prevent BVIN from progressing unmanaged and becoming severe and likely chronic.

Unfortunately, there is no gold standard tool for how patients with cancer including those with HL should be routinely assessed for the detection and grading of CIPN, including even in clinical trials [104]. Currently, in both research and clinical practice, clinicians usually use a standardised toxicity scale that quickly assesses and scores patient’s symptoms and difficulty with everyday tasks to determine BVIN grading: The National Cancer Institute (NCI) Common Terminology Criteria for Adverse Events (CTCAE) (https://ctep.cancer.gov/protocoldevelopment/electronic_applications/ctc.htm, accessed on 31 October 2021). Grade 1 refers to mild symptoms or the presence of subclinical neuropathy, grade 2 includes moderate symptoms that limit instrumental activities of daily living (ADLs), and grade 3 as severe symptoms limiting self-care ADLs. Grade 4 is defined as PN with life-threatening consequences and urgent intervention indicated. Of note, distinction between PN of grades 2 and 3 may vary depending on a patient’s age and status as it relates to performance, profession, family, housing, and leisure time activities. In the setting of BVIN, clinically relevant PN is usually considered ≥ grade 2. Importantly, motor neuropathy should be routinely addressed separately from the sensory scale, in a specific motor scale. Symptoms due to autonomic dysfunction such as hypotension, urinary retention, erectile dysfunction, or constipation should be rated according to specific symptoms.

However, CTCAE cannot be suggested solely as a clinician-based grading outcome measure for the evaluation of CIPN, including BVIN [104]. The CTCAE scale is known to lack interobserver reliability and responsiveness [105,106,107] and possibly be poorly informative regarding neurologic impairments [104]. Moreover, CTCAE is based on subjective patient reporting and over-emphasises “positive symptoms” such as tingling and pain sensations whilst overlooking “negative” symptoms, including loss of sensation, troubles balancing or even weakness [79]. In this setting, using specific scales to be more precise in diagnosing and grading the progression and severity of BVIN has become increasingly recommended [21]. Amongst them, the Total Neuropathy Score (TNS) (^©^Johns Hopkins University) is a considerable tool of choice. TNS^©^ is a composite scale that includes clinical a neurologic and neurophysiologic examination combined with patient symptom reporting. It has demonstrated strong reliability and validity for CIPN assessment. TNS evaluates the extent of symptom and sign progression in the limbs, albeit not pain severity. Several variants of the TNS such as modified TNS (TNSm); clinical TNS (TNSc); reduced TNS (TNSr); and, more recently, nurse TNS (TNSn) have been used to evaluate CIPN onset, serving as a validated clinical tool in this setting [104,108], BVIN included. A good correlation between TNSc and CTCAE was reported in a series of 36 patients with MF/Sezary syndrome receiving BV prospectively evaluated, with a grade 2 PN occurring around a TNSc of 6–12 score [79]. TNSn, which is calculated from five of the seven items in the TNSc, can be considered as a reliable, alternative, and objective clinical assessment where strength and reflex testing is removed. It can be useful if a more extended neurologic examination is not possible, as it can be done by a trained healthcare professional involved in the multidisciplinary approach of BVIN [103,104,109,110]. Very recently, the cut-off values for a relevant change defined as minimal clinical important difference (MCID) of TNS have been reported [104], supporting TNS implementation in clinical trials of patients treated with BV. Corbin et al. suggested that TNSc may allow for earlier detection of BVIN before the condition becomes clinically significant. However, formal comparisons between TNSc and CTCAE grading for BV dose modification are lacking [79] and the role of TNS responsiveness as an outcome measure in the clinical trial setting should be further explored.

Clinician-graded scales alone are insufficient to assess CIPN adequately. Discrepancies between clinicians’ assessment of CIPN and patient-reported outcome measures (PROMs) can be frequently observed. Systematic and standardised assessment of symptoms may be helpful by the use of several PROMs (i.e., EORTC-CIPN20 and FACT/GOG-Ntx). MCID has been reported for FACT/GOG-NTX [111] and EORTC-CIPN20 [112]. For example, numbness and tingling items from patient-reported outcomes (PRO) of the CTCAE (PRO-CTCAE)—which asks patients to rate the severity of these PN symptoms and the degree to which they have interfered with daily activities—can be useful to monitor BVIN. The PRO-CTCAE can be downloaded for free on the NCI website (see https://healthcaredelivery.cancer.gov/pro-ctcae/pro-ctcae_spanish.pdf, accessed on 31 October 2021). In older patients, the use of comprehensive geriatric assessment outcomes, including before treatment, are highly recommended [113].

Integrating CIPN assessment tools in the clinical setting requires time, and it is important to coordinate study assessments with clinical visits whenever possible to minimise the burden on patients [114,115]. Along this same line, a published research survey examining patient preferences for the design of a CIPN assessment tool in neurotoxic chemotherapy-treated patients with cancer revealed a preference for a 30 min addition to routine clinic time to accommodate a detailed, proactive CIPN assessment. Such an assessment would include objective and instrumental assessment tools that would favour shared decision-making processes with the treating team [116]. In summary, a combination of physician-based methods, varying PROM scales, and sufficient time to properly assess PN are needed to comprise the most reliable approach to measure BVIN through valid means [117].

### 6.2. Proper Management of BVIN Requires a Multidisciplinary Approach

#### 6.2.1. Dose Adjustments

BV is usually administered on an outpatient basis. The recommended dose-and-administration schedule for BV in patients with either relapsed classical HL or HL with a high risk of progression after auto-HSCT is an intravenous infusion of 1.8 mg/kg for 30 min every three weeks. For previously untreated patients with classical HL receiving chemotherapy, the recommended dose-and-administration schedule for BV is 1.2 mg/kg up to a total of 120 mg every two weeks for a maximum of 12 doses [4,5]. For re-treatment, the starting BV dose should be either 1.8 mg/kg or the last tolerated dose [62]. Once treatment begins, patients should be assessed for PN at every visit; the cumulative dose of BV is the most important predictor of severe BVIN. The only current strategies to address BVIN worsening are dose delay, dose reduction, or treatment discontinuation according to CTCAE grading scales for sensory, motor, and autonomic symptoms. BV adjustments or delays may occur with the onset of grade 2 neurotoxicity. Currently, for PN of grade 2 or 3, BV dosing should be stopped until PN improves to grade 1 or baseline, after which the patient can restart treatment at a lower dose of 1.2 mg/kg. BV should be permanently discontinued in cases of grade 4 PN (https://pdf.hres.ca/dpd_pm/00045998.PDF, accessed on 31 October 2021). Of note, some differences in management have been observed in literature. In some trials patients who presented grade 2 neuropathy had a delay/reduction in BV dosing, whilst patients who presented grade 3 neuropathy stopped receiving therapy [6]. In real-life experience, however, a BV dose reduction to 1.2 mg/kg was recommended for grade 3 toxicity and only interrupted when grade 4 toxicity occurred [47,50].

BV treatment had a manageable neurotoxicity in patients with HL participating in clinical trials and studies within a real-world setting (Table 1). Dose modification is a successful, preventative strategy in managing patients with BVIN, with supporting evidence by pharmacokinetic analyses demonstrating that the probability of PN onset is associated with BV exposure [64,118]. Reducing doses thus lowers peak plasma concentration and minimises toxicity [71]. Clinical trials have shown that BVIN signs and symptoms can be reversible upon dose adjustment or therapy withdrawal. In detail, the AETHERA trial resulted in dose modifications in 31% of patients and treatment discontinuation in 23% of patients. More than half of the 51 patients with PN (57%) who required dose modifications completed all 16 cycles of BV therapy [6]. By moderating BV treatment, clinicians are able to both optimise treatment whilst limiting neurotoxicity.

#### 6.2.2. Patients

Patient preferences with regards to potential toxicities and treatments are important in relation to decision-making processes regarding treatment [116]. When BVIN occurs, patients and clinicians may face the dilemma by choosing whether to reduce chemotherapy or even stop it altogether to prevent a worsening pf PN. Clinicians should discuss the appropriateness of dose delay, dose reduction, or chemotherapy cessation (or substitution with agents that do not cause PN) with patients who develop intolerable neuropathy and/or functional nerve impairment [119]. Although patients and physicians significantly prefer first-line treatments that offer a lower risk of PN [120], a recently published and large cross-sectional survey including 381 patients with HL from Germany, France, and the United Kingdom showed a willingness by patients to accept side effects for an efficacious treatment. Patients preferred first-line HL treatments that conferred a lower risk of HL returning and longer survival. Indeed, they were willing to accept increased side effects including PN if such side effects meant an increase in chances of survival [120]. Of note, in a safety analysis of BV performed in the phase III AETHERA trial exploring HL in a post-transplant consolidation setting, patients with PN-related dose modifications had 4 year progression-free survival (PFS) comparable with patients without such adjustments (60% (95% CI, 43% to 74%) or 65% (95% CI, 50% to 77%), respectively) [121]. To apply a judicious implementation of dose delays and/or reductions, there is a patient-centric decision framework that clinicians can use to assess an individual patient’s current and possibly future CIPN severity and compare that to their maximum tolerable severity. The framework can help clinicians determine whether they should continue, delay, decrease, or discontinue neurotoxic chemotherapy [122].

#### 6.2.3. Multidisciplinary Approach

**Figure 2:**
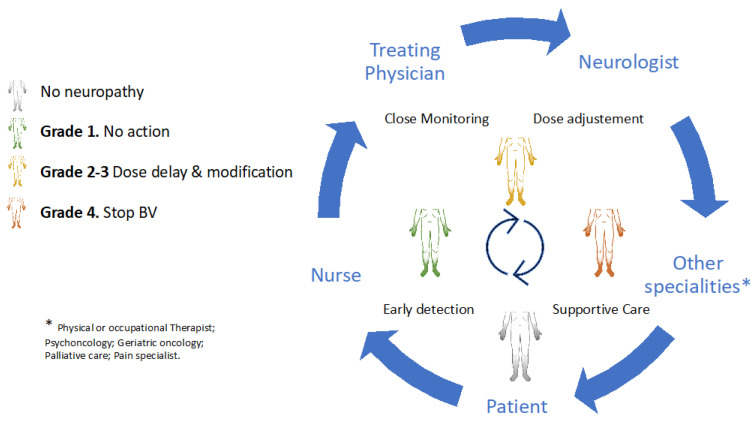
A multidisciplinary approach in brentuximab-induced peripheral neurotoxicity.

Early detection and timely multidisciplinary intervention are cardinal in maintaining the safety and functional independence of patients with HL. Temporary or permanent BV discontinuation should be considered depending on the severity of neurologic impairment, oncologic status, and the availability of alternative antitumoural therapies within the ideal setting of a multidisciplinary team. Collaborative strategies that involve experienced haematologists, oncology nurses, nurse practitioners, neurologists, and other specialities (e.g., cancer rehabilitation physiatrists, or physical or occupational therapists) and patients are of the utmost importance in these scenarios (Figure 2).

**Figure 3:**
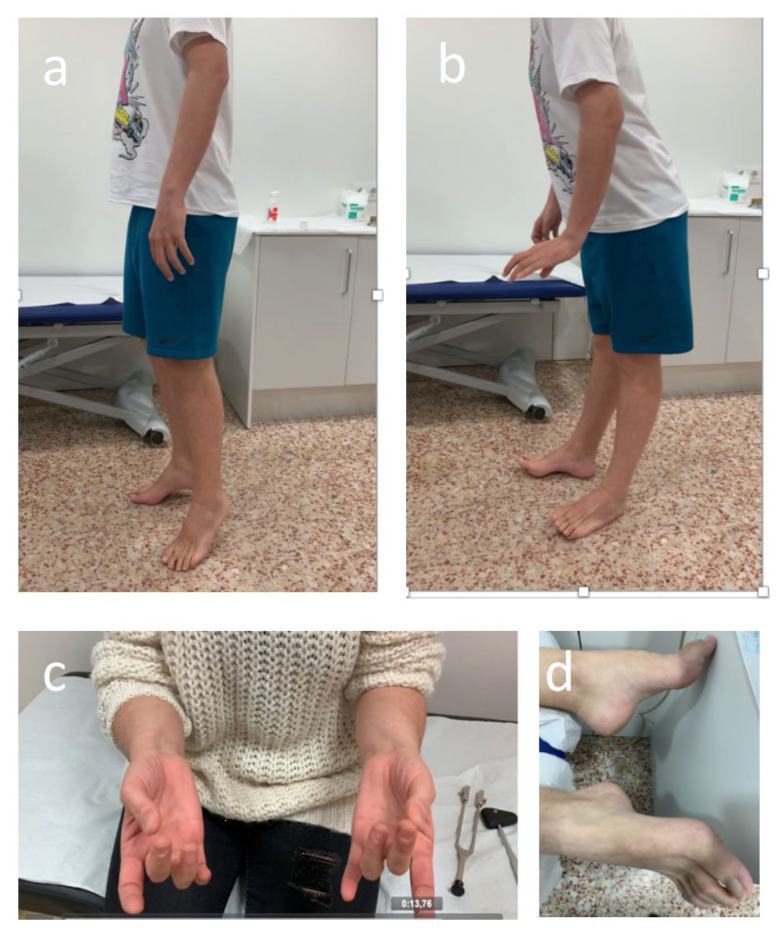
Abnormal ankle dorsiflexion strength test (**a**,**b**), clapping (**c**), and cavus feet (**d**) in patients with sensory-motor BVIN.

Throughout treatment, patients should be monitored for signs and symptoms of neurotoxicity. By encouraging open communication and speaking with patients and caregivers about the possibility of BVIN onset and the benefits of early symptom reporting, BV adherence and effective management of PN may improve [103,109]. Having expertise relevant to BVIN management should form part of nurses or assistant physicians’ routine practice with patients with HL. A comprehensive assessment should with such healthcare professionals observing patients as they walk into the examination room in order to evaluate any abnormal gait or difficulty with balance. Similarly, it is important to see whether patients wear slip-on clothing or slip-on footwear, such as slippers or flip-flops, and observe patients as they button clothing, tie shoelaces, and pick up small objects (e.g., coins or paper clips) [109,110]. The clinical evaluation by the treating physician assistant or nurse practitioner should be followed by screening for related sensory impairments. This includes asking patients whether they have experienced any sensory symptoms (i.e., numbness, tingling, pain, “pins and needles,” etc.) and to use a number of neuropathic sensations descriptors (i.e., resembling a burning or electric shock sensation, or experiencing “ice-cold” or “fallen asleep” hands or feet) that may help patients recognise BVIN related symptoms. Healthcare professionals should also systematically ask patients if they have any difficulty completing everyday tasks, such as using a fork, knife, or pen; typing; opening a jar; or walking. Fine-motor activities, including buttoning shirts, fastening jewellery, pulling up zippers, putting on earrings, using remote controls, painting, knitting, sewing, and turning pages in a book, can become challenging to perform [101]. Furthermore, healthcare professionals could also assess physical function as it relates to PN. Screening for motor-related impairments affecting hand grip, wrist extension, ankle dorsiflexion strength, and gait is important in detecting motor PN early (Figure 3).

Nurses are of paramount importance in educating patients with HL about BVIN risk-reducing measures and management strategies for PN. Fall precautions (i.e., adequate lighting, or the removal of tripping hazards) and hand and foot care (i.e., gloves whilst working with sharp, hot, or cold objects, and proper-fitting shoes) comprise some priorities in safety education. Several other strategies in assessing and educating patients who are at risk for or are already experiencing CIPN have been recently reviewed [103].

To provide quality multidisciplinary care, incorporating neurologic assessments and a neurologist’s input is also important. Such an approach ensures the best outcomes for this type of neurotoxicity. Early identification of symptoms will allow for appropriate and quicker referral to a neurologist. A physical examination by a neurologist that objectively assesses the patient’s strength, sensation, reflexes, and gait should determine if moderate-severe BVIN is developing. The results obtained from this examination could help haemato-oncologists in the decision-making process. In detail, to catch the onset of PN, the most prominent clinical exam finding appears to be a vibratory sensory loss, which indicates extensive sensory fibre damage [21,79,85]. It can be easily assessed using a semi-quantitative tuning fork (Figure 3c). In the setting of typical forms of BVIN presentation, referral to a neurologist and NCS can prove useful in confirming the diagnosis and evaluating the PN type and severity. In fact, all patients developing motor signs whilst receiving BV should undergo a neurologic assessment and NCS to determine the presence of demyelinating abnormalities which could prompt the need for immune therapy [84]. Furthermore, continual neurologic assessments in patients with HL receiving BV treatment could allow for a longer duration of BV therapy, and thereby possibly extended oncologic benefits. Importantly, although the most frequent neuropathies in HL are drug-induced, rarely is the PN system ever involved at any stage in the course of this lymphoproliferative disease due to other reasons including paraneoplastic, radiation-induced, or lymphoma dissemination to the peripheral nervous system [98,99]. Time course and the link with treatment initiation will be extremely helpful in the diagnosis. An early referral to neurology department should be advised in those patients presenting with atypical or rapidly progressive features [123]. Finally, sequential electro diagnostic work-up has proven useful in identifying subclinical BVIN [21] and monitoring PN involvement with other chemotherapy agents [124,125]. However, the role of NCS in monitoring BVIN requires further investigation.

Physical therapy (PT) might be a helpful intervention in the treatment of CIPN [126]. Symptoms due to BVIN can lead to functional problems and disability, including altered gait, sensory disturbance, and an elevated risk of falls [88]. In this setting, a treating physician or neurologist may consider PT and/or occupational therapy approaches for patients with physical dysfunction or balance troubles [119]. Exercise and physical activity-based interventions including balance, sensorimotor, or fine motor skill training have shown to be promising in reducing the burden of existing CIPN symptoms and potentially preventing toxicity. Exercise may be beneficial for patients with HL receiving BV, even though evidence that exercise rehabilitation strategies may be effective in patients with CIPN is limited to date and no definitive data to conclude that exercise interventions ameliorate established CIPN are available. With respect to the latter, this is in part due to small sample sizes, variable outcome measure selection, and little data on how exercise affects CIPN mechanistically [127,128]. Similarly, there is a growing body of literature to suggest that exercise could even prevent CIPN [129]. Sensorimotor training showed to improve balance control, physical performance level, and mobility during therapy in patients, especially those with lymphoma [130].

Importantly, HL is generally a curable haematological malignancy [63]. Some patients with HL receiving BV will experience persistent or long-term PN. Proactive referrals to other supportive resources and multidisciplinary care can help to optimise outcomes for these patients [131]. Feelings of frustration, embarrassment, isolation, anxiety, depression, and loss of purpose are common in patients with CIPN given the forced need to forgo usual activities [132]. Cognitive behavioural therapy, palliative care, social work (if financial toxicity is an issue), and support groups could help patients cope and manage their BV-induced toxicity and its accompany mental conditions. Psychologic interventions (e.g., cognitive behavioural therapy or meditation/mindfulness) and other non-pharmacologic approaches seem promising. However, large, randomised trials are necessary to show efficacy of such strategies in relief support from CIPN [110].

#### 6.2.4. Pharmacologic Treatment of BVIN

Pharmacologic management of BVIN focuses predominantly on symptomatic supportive care. To date, duloxetine, a serotonin–norepinephrine reuptake inhibitor, is the only pharmacologic agent recommended in BVIN by clinical practice guidelines provided by the American Society of Clinical Oncology [119]. In a randomised, blinded, placebo-controlled crossover trial conducted amongst 231 individuals with chronic moderate-to-severe paclitaxel- and oxaliplatin-induced PN, one-third of patients experienced a clinically significant (30%) reduction in CIPN pain severity by week 6 [133]. Duloxetine showed to be effective in decreasing pain and relieving non-painful symptoms such as numbness and tingling [133]. In our experience, female sex and short-term CIPN were independently associated with a favourable response to duloxetine [134]. Unfortunately, up to one-third discontinued treatment due to duloxetine-related adverse events observed by our team [134] and other investigators [135]. In relation to other treatments, such as tricyclic antidepressants, gabapentin, pregabalin, or topical amitriptyline/ketamine/baclofen for treating established CIPN, despite symptomatic benefit being observed in some patients, no recommendations can be made from the current ASCO CIPN guidelines [119]. Importantly, timely symptom management of positive sensory symptoms or neuropathic pain might influence dose delay/reduction and the prolonged use of BV in some patients. In these cases, the need for detailed neurologic monitoring to avoid misdiagnosis of severe motor neuropathy is advisable.

IGIV or plasma exchange (PE) to remove residual drug has been tested in some patients [84], with partial improvement of motor strength following PE sessions [85]. The role of corticosteroids in severe motor BVIN has been suggested after the clinical and neurophysiologic improvement of DLs and proximal CMAP amplitudes observed after corticosteroid treatment. However, the potential confounding effect of discontinuing the offending agent cannot be ruled out [84,85].

## 7. Prognosis and Long-Term Outcomes of BVIN

BVIN is usually reversible following BV withdrawal. Relatively long-term safety evaluation studies of BV therapy investing the incidence and recovery of BV-related PN after the completion of neurotoxic chemotherapy are scarce. According to the literature, most patients tend to experience partial or complete resolution of symptoms (Table 1). In phase II and III trials, resolution or improvement was reported in 80–85% [3,6,9], with complete PN resolution in 48–50% of patients [3,9]. In these pivotal studies carried out in North America and Europe, the median time to improvement or resolution was 9.9–13.2 weeks [3,9]. Conversely, resolution of symptoms was not observed in most patients at the end-of-treatment visit in a phase I/II trial, including a median of 16 (4–16) treatment cycles [29]. Differences in the time of follow-up for neuropathy events could provide an explanation for this finding. A continued improvement of PN with BV in terms of severity according to CTCAE has been observed. A rate of PN resolution at 60 months after the end of treatment was 85% in the AETHERA trial [121]. In a real-life experience, a retrospective, multicentre Italian study including 234 patients with R/R HL reported resolution or improvement of PN in 90% of patients with a median time to improvement of at least 12 weeks [50]. Importantly, the lack of a formal neurologic assessment is a main limitation in both clinical trial cohorts and real-life studies. Although resolution of symptoms can be observed in many patients during follow-up, it does not mean that PN disappears; ongoing asymptomatic grade 1 findings may be more frequent than described [24]. This is why a lesser extent of overall resolution is perhaps usually observed in neurologically monitored patients. In the few studies including a comprehensive neurologic follow-up, only 44% of HL cases experienced PN improvement after a median follow-up of 12 (3–19) months [21]. In the MF population, the median time to improvement or resolution of any grade of PN was 53 weeks from the last BV dose, and only half (52%) of all patients showed overall improvement or resolution of PN by the end of the follow-up period. In detail, at 12 and 24 months, 47% and 66% of patients had improved, respectively. A shorter time of PN amelioration or resolution of 30 weeks was observed amongst patients with clinically significant BVIN (≥grade 2), with a rate of improvement of 51% and 74% at 12 and 24 months, respectively [79]. The only grade 3 event observed in a phase I trial returned to grade 1 after approximately four months [24]. During recovery, restoration of upper limb reflexes and improvement in ambulation and muscle strength were described [74]. Moreover, an increase in nerve CVs and amplitudes in neurophysiologic findings revealing an objective improvement were reported [74], as observed in our experience (Figure 1). Fargeot et al. reported that nine patients (82%) improved during follow-up, although none of them returned to their baseline status. At the last follow-up, a median of 15 months after neurologic onset, all patients had mild-to-moderate residual deficits, although one patient (9%) still required help for walking [84]. Motor neuropathy may have a longer median time to improvement or resolution. Of note, a case report of a grade 3 motor PN obtained almost clinically complete resolution within 30 weeks after drug interruption [85].

After the last dose of BV, BVIN symptoms can peak within the first weeks of treatment and then fall gradually over time. BVIN can even appear or worsen after therapy withdrawal in what is known as the “coasting effect” [74,79], defined as a worsening or development of CIPN up to three months after neurotoxic chemotherapy completion. It is typically observed with platinum agents and described with microtubule-disrupting agents such as vincristine and paclitaxel [136]. Corbin et al. reported that 12 of 18 (66.6%) patients with MF who developed clinically significant BVIN worsened after the last BV cycle [79].

Rates of resolution or improvement in PN in older patients included in the ECHELON-1 trial appeared similar to those in older patients with cHL treated with A+AVD and ABVD (80% vs. 83%, respectively). However, observed rates of complete resolution were lower compared to those in younger (<60 years) patients (56% vs 74%) [93]. Interestingly, no differences in time to resolution/improvement of PN in older patients with risk factors such as hypothyroidism or DM were observed (a median time of 9.8 weeks vs. 8.9 weeks for patients without risk factors) [90].

PN may affect the lives of patients with HL. In a QoL analysis of the AETHERA study, patients who experienced PN demonstrated lower EuroQol Group European Quality of Life 5-dimensional instrument (EQ-5D scores) than patients in the placebo arm. These were not statistically different and did not reach the MID at any time point [137], revealing that there were no significant differences in mean EQ-5D scores between patients with and without PN within the BV arm at any time [63]. Recently, 303 survivors of HL at a median of 16 years after diagnosis with a median follow-up of 16 years reported significantly higher neuropathy symptom burden than controls [138]. However, no independent association between symptoms and disease or treatment-related variables were found; moreover, neither the type nor the number of chemotherapy cycles given were identified in this study. Authors suggested that PN symptoms may be related to factors other than neurotoxic chemotherapy [138].

## 8. Conclusions

Although treatment-induced PN is not a new problem in HL therapy, BVIN frequency and its potentially disabling nature highlight the need for close monitoring in everyday clinical practice during and after completion of BV treatment. Early detection and intervention by dose adjustments are critical for a better outcome. A multidisciplinary approach and expertise in BVIN will contribute to improving care for the increasing number of patients with HL undergoing treatment with BV.

## Figures and Tables

**Table 1:** Incidence and severity of BV-induced PN in R/R cHL in the adult population. Publications released worldwide reporting on PN toxicity and including > 20 patients with R/R HL are included.

Author Year	Study *N*	Schedule	Age Median, (Range)	Cycles· Median, (Range)	Overall PN	Grade ≥3 PN	Cessation due to PN	PN Onset	PN Evolution (Median Time)
BV MONOTHERAPY IN RELAPSE/REFRACTORY HL CLINICAL TRIALS									
Younes 2010	Phase I	SA	36 y	-	36%	1 pt	3 pt	9 w	63% CR
[24]	45 (42 HL)		data					(3–24)	
Fanale 2012	Phase I	SA	33	4	73%	S: 6 pt	8 pt	Any: 6.1 w	G2: Time R/I: 12.1 w
[25]	44 (38 HL)	Weekly dose	(12–82)	(1–12)		M: 3 pt		G3: 25.9 w	G3: Time I: 21.6 w
Gopal 2012	Phase II	SA	32	8	52%	*2 pt*	5 pt	-	54% R/I
[26]	25		(20–56)	(1–16)					
Younes 2012 [3]	Phase II	SA	31	9	55%	8 %	9 pt	Any: 12.4 w	80 % R/I; 50% CR
Chen 2016 [27]	102		(15–77)	(1–16)				G2/327.3– 38.0w	Time R/I: 13.2 w
Forero–Torres 2012 [28]	Phase I 20	SA * 9 pt weekly	31.5 (12–87)	-	45%	0%	0 pt	-	-
Ogura 2014	Phase I/II	SA	41	16	60%	0 %	-	11.3 w (0.3–48.9)	Only resolved 1 pt
[29]	20		(22-88)	(4-16)					
Chen 2015	Phase II	SA	34	4	52%	0 %	0 pt	-	-
[30]	37		(11-67)	(1-4)					
Moskowitz	Phase III	SA	33	15	67%	S: 10%	38 pt	13·7 (0·1–47·4) w	90% R/I; 73% CR
[6,7]	329		(18–71)	(1–16)		M: 6%	(23%)		Time R/I: 37.6 w
Walewski 2018	Phase IV	SA	32	7	35%	3%	1 pt	9.4 w (0.6–39.1)	57 % CR
[31]	60		(18–75)	(1–16)					
Stefoni 2020 [32]	Phase II 20	SA	73 (61–86)	7 (1–16)	33%	1 pt	3 pt	PN starting from cycle 2	-
Kuruvilla 2021	Phase III	SA	35	12% > 16	18%	3 %	8 pt	-	-
[33]	153		(25–80)	doses					
Song 2021	Phase II	SA	30	10	47%	0%	3 pt	Any: 10.6(0.3–45.4) w G2: 11.9 (0.3–45.4) w	55% R/I
[34]	39 (30HL)		(21–64)	(2–16)					
BV IN COMBINATION WITH CHEMOTHERAPY IN R/R CLINICAL TRIALS									
Moskowitz 2015	Phase II	SA seq**	31	2	49%	0 %	0 pt	-	-
[16]	45	Weekly After ICI	(13–65)						
O’Connor 2018	Phase I /II	C	38 (25–70)	I: 6	32%	-	-	-	-
				(1–6)					
[4]	I (28); II (37)	+Benda	34 (18–72)	II: 5					
				(2–6)					
LaCasce 2018/2020	Phase I/II	C	36	10	54.4%	3.6%	7.3%	-	63% R/I
[35,36]	53	+Benda	(19–79)	(1–14)					R/I: 3 w (0.4–37)
Herrera 2018	Phase I/II	C	36	4	20%	1 pt	1 pt		
[37]	61	+ Nivo	(18–69)	(1–4)					
Garcia-Sanz 2019	Phase I/II	C	36	Up to 7	22%	-	3 pt	-	All resolved
[17]	66	BRESHAP	(18–66)	C					
Broccoli 2019	Phase II	C +Benda	38	4	1.8%		0 pt	-	100% R
[32]	40	BBV	(20–59)						
Diefenbach 2020	Phase I/II	C	33–40	7	52%	1 pt	-	-	-
[38]	64	+Ipi/Nivo	(26–51)	(4–12)					
Kersten 2021	Phase II	C	29	3	32.7%	0 %	0 pt	-	100 % R
[39]	55	BV-DHAP	(19–71)						
Lynch 2021	Phase I/II ***	C	31	4 doses	36%	2%	-		
[40]		BV-ICE	(28–45)						
BV MONOTHERAPY IN R/R HL RETROSPECTIVE STUDIES									
Rothe 2012	Retrospective	SA	35	7	31%	0 %	0 pt	-	-
[41]	45			(1–12)					
Gibb 2013	Retrospective	SA	41.5	5.5	-	3 pt	-	-	-
[42]	24		(21–78)	(1–13)		(12.5%)			
Zinzani 2013	Retrospective	SA	27.5	8	21.5%	5 pt	3 pt	-	90 % R/I
[43]	65		(12–66)	(3–16)					Time R/I: 12 w
Graciaz 2014	Retrospective	SA	35	5	-	1 pt	0 pt	-	-
[44]	24		(20–60)	(2–8)					
Yang 2014	Retrospective	SA	30	5	-	0 pt	-	-	-
[22]	22		(16–57)	(1–18)					
Salihoglu 2015	Retrospective	SA	26	7	32.7%	2 pt	1 pt	-	-
[45]	58		(13–62)	(2–18)					
Monjanel 2014	Retrospective	SA	35	7	11.1%	0 %	-	-	-
[46]	45 (32 HL)		(20–69)	(1–16)					
Perrot 2016	Retrospective	SA	30	6	29.3%	2.3 %	-	PN peaking	-
[47]	240		(14–78)	(1–16)				at cycle 7	
Brockelmann 2017	Retrospective	SA	70	8	9.6%	-	-	-	-
[48]	136			(6–15)					
Eyre 2017	Retrospective	SA	32	4	9%	2 pt	-	-	-
[49]	99		(13–70)	(1–9)					
Pellegrini 2017	Retrospective	SA	35.4	6	-	7.2%	-	-	90% R/I
[50]	234		(18–79)	(1–16)					Time R/I: 12w
Zagadailov 2018	Retrospective	SA	50.5%	7.5	8.7%	-	17.6%	-	-
[23]	196		>45 y	(5–11)					
Pavone 2018	Retrospective	SA	34	4/8	50%	7%	-	-	-
[51]	70		(15–84)	(1–16)					
Clairivet 2018	Retrospective	SA	43.2	5	33.3%	0%	-	-	-
[52]	39		(14–82)	(1–23)					
Tien 2019	Retrospective	SA	28	5.5	20%	-	-	-	-
[53]	20		(15–85)	(1–19)					
Král 2019	Retrospective	SA	30.5	7.5	36.2%	0%	0 pt	-	-
[54]	58		(20–53)	(3–16)					
Kort 2020	Retrospective	SA	25.5	4	-	0%	0 pt	-	-
[55]	20		(18–61)	(3–4)					
Izutsu 2021	Retrospective	SA	62	5.5	40.7% S	8.2% S	23 pt	Within 9 w in 61%	-
[56]	182		(14–93)		5.0% M	3.9%M		Within 27 w in 90%	
Akay 2021	Retrospective	SA	31	8	21%	11%	9 pt	-	-
[57]	75		(18–65)	(3–15)					
BV IN COMBINATION WITH CHEMOTHERAPY FOR R/R HL RETROSPECTIVE STUDIES									
Ianitto 2020	Retrospective	C	34	48%	-	11%	-	-	-
[58]	47	BVB	(18–76)	6 cycles					
Damlaj 2020	Retrospective	C	22	15	35%	5%	20%	-	100% R/ I
[59]	20	(Various)	(15–47)	(5–16)					
Pinczés 2020	Retrospective	C	-	3	12.2%	2.4%	-	-	-
[60]	41	BVB		(1–16)					
Uncu Ulu 2021 [61]	Retrospective 61	C BvB	33 (18–76)	4 (2–11)	NR	6.5%	4 pt	-	R in 2 pts, 2 pt G3 PN was stable

* Weekly schedule; ** BV 1.2 mg/kg on days 1, 8, and 15 for two 28-day cycles; *** BV on days 1 and 8 at either 1.2 mg/kg (dose level 1) or 1.5 mg/kg (dose level 2) intravenously (capped at 150 mg) with standard dosing of ICE on days 1–3 (ifosfamide 5 g/m^2^ plus mesna 5 g/m^2^ intravenously) over 24 h on day 2; carboplatin AUC 5 on day 2: and etoposide 100 mg/m^2^ on days 1–3 for two 21-day cycles. W: weeks; SA: single agent refers to 30 min infusion of BV at a dose of 1.8 mg/kg of body weight every 3 weeks; C: combination; B: bendamustine; ICI: immune checkpoint inhibitor; R: resolution; I: improvement; CR: complete resolution; S: sensory; M: motor; PN: peripheral neuropathy: pt: patients.

## Data Availability

No new data were created or analyzed in this study. Data sharing is not applicable to this article.
